# Clinical and economic impact of a one-year treatment with omalizumab in patients with severe allergic asthma within a drug programme in Poland

**DOI:** 10.1186/s12890-018-0610-z

**Published:** 2018-03-16

**Authors:** Karina Jahnz-Różyk, Joanna Lis, Marta Warchoł, Aleksandra Kucharczyk

**Affiliations:** 1 Department of Internal Diseases, Pneumonology, Allergology and Clinical Immunology, Military Institute of Medicine, Szaserów 128, 04-141 Warsaw, Poland; 2 Sanofi-Aventis SP. z o.o, Bonifraterska 17, 00-203 Warsaw, Poland

**Keywords:** Severe asthma, Omalizumab, Drug programmes in health care system in Poland, Cost of treatment

## Abstract

**Background:**

Allergic asthma is the most prevalent phenotype of severe asthma where treatment with omalizumab (OMB) has been proven to be particularly beneficial. In Poland, OMB therapy is available and reimbursed within a drug programme where strict inclusion and exclusion criteria are defined.

The objective of this study was to present a descriptive analysis regarding the trends in outcomes (clinical, quality of life, costs) among a cohort of patients who satisfy inclusion criteria for the initiation of OMB treatment and who successfully responded to OMB according to a set of objective criteria.

**Methods:**

A retrospective analysis of data collected during the 52 weeks of OMB treatment was carried out. The study population was adolescents and adults with severe allergic asthma that was uncontrolled despite a combination of high-dose inhaled corticosteroids (ICS)/long-acting beta-agonists (LABA) and/or other controllers (leukotriene receptor antagonists (LTRA), sustained-release theophylline, and short- or long-acting muscarinic antagonists (SAMA/LAMA), who were the first to finish the one-year treatment. A clinical and cost analysis for patients included in the programme was conducted comparing the one-year pre-treatment period to the one-year treatment period outcomes.

**Results:**

Data of 85 patients who completed the first year of therapy were reviewed and analysed. Add-on OMB treatment resulted in a median decrease in exacerbation rate of 66% relative to the baseline and a reduction in oral steroid (OCS) dose by an average of 7.7 mg. At the end of the 52 weeks of therapy the changes in the quality of life questionnaire (AQLQ) and the asthma control questionnaire (ACQ) scores were 1.86 and 1.45 points, respectively. The mean cost of asthma treatment increased by an average of 15,979 EUR per patient per year (baseline period – 802 EUR/patient/year; OMB treatment – 16,781 EUR/patient/year). The cost to avoid one exacerbation was 17721 EUR.

**Conclusion:**

The clinical outcomes for the observed subset of patients were highly improved. At the same time, costs of the treatment increased, mainly due to the high OMB costs. Other costs associated with a lower number of hospitalizations and ED and office visits and a reduction in OCS dose decreased. These descriptive data can be used for further investigation in defining patients who benefit the most from OMB treatment in clinical practice.

## Background

Although severe asthma applies to only 5 to 10% of patients with asthma, a substantial portion of the health care costs is associated with this phenotype of the disease [1–3]. Only a small part of the cost is a result of diagnostic tests, which must be performed to differentiate severe asthma from other chronic diseases and to assess the impact of other diseases on the course of asthma. The greatest expenses are associated with treatment - an inadequate response to therapy, an inability to achieve sound asthma control and an increased risk of exacerbation – all of which result in additional high costs that affect health care utilization (HCU).

With the emergence of new data on the pathogenesis of asthma, the diversity of clinical presentations and the response to treatment, it is increasingly believed that severe asthma is a heterogeneous disease comprising discrete phenotypes that differ in pathogenesis, genetic background and clinical outcome [4, 5]. One of the phenotypes is allergic asthma.

OMB is a recombinant humanized monoclonal anti-IgE antibody designed to treat IgE-mediated disease by reducing the plasma concentration of free IgE antibodies. The efficacy and safety of OMB in severe persistent allergic asthma have been confirmed in numerous multicentre clinical trials [6, 7] where OMB as an add-on therapy reduced the number of asthma exacerbations and concomitant medication burden and improved symptom severity and quality of life (QoL) compared to the use of standard therapy alone.

The Global Initiative for Asthma (GINA) guidelines [8] recommended that OMB be considered an adjunctive therapy for patients with moderate or severe allergic asthma that is uncontrolled on step 4 of treatment. Per GINA, a stepwise approach is needed for adjusting asthma treatment to achieve good symptom control and minimize the future risk of exacerbations, fixed airflow limitation and medication side-effects. The choice of therapy may, however, be affected by the cost of treatment.

Innovative therapies (primarily biological therapies) in Poland are reimbursed within drug programmes [9]. A drug programme is “guaranteed compensation, including therapies with innovative, expensive active substances which are not financed by other guaranteed benefits. The treatment is carried out in selected disease entities and includes a strictly defined group of patients”. The advantage of carrying out treatment within drug programmes is more detailed qualification of patients recruited for treatment, based on well-described inclusion and exclusion criteria [10]. Check points allow, in turn, for a more detailed assessment of the efficacy, safety, and cost of the therapy.

Patients suffering from severe allergic asthma in Poland are treated within the drug program “Treatment of allergic, IgE-mediated asthma with omalizumab” [10]. This study evaluated the clinical outcomes and costs of add-on OMB therapy for severe allergic asthma in Poland based on a group of 85 patients who were the first to finish the one-year treatment.

## Methods

### Study design and data dources

The Drug Program Registry is a multi-centre, single-arm, open-label, observational registry. Patients with a diagnosis of severe IgE-dependent allergic asthma who met the inclusion and did not meet any of the exclusion criteria of the Polish Drug Program (Table 1) and who were treated with OMB for one year (in 2013 and 2014) at 36 centres in Poland were eligible for the assessment. Patients were recruited for the study using an electronic database, which is owned by the Polish Ministry of Health. The authors obtained the written consent of the Ministry for the use of the data, in accordance with regulations for the publication of patient data. The analysis included all patients subsequently entering this registry. All of them provided informed consent before each OMB administration.

**Table 1:** Inclusion and exclusion criteria in Polish drug programme with OMB

Inclusion Criteria	
1	Adult and adolescent patients with severe, uncontrolled allergic asthma (per GINA guidelines), allergy to perennial allergens confirmed with positive results of skin prick or specific IgE tests
2	The need to use high doses of ICS (> 1000 mcg of beclomethasone dipropionate (CFC) per day or equivalent) in combination with a second controller (i.e. LABA, LTRA, theophylline)
3	Frequent use of OCS in the past, including the last 6 months
4	Total serum IgE levels 30–1500 IU / ml
5	Unequivocal in vitro reactivity to a perennial allergen in patients with total serum IgE (tIgE) levels below 76 IU/ml
6	The fulfilment of at least three of the following criteria: a) Uncontrolled asthma symptoms (lack of asthma control in ACQ > 1.5 points) b) Three or more episodes of exacerbations a year requiring systemic corticosteroids or increase their current dose. c) Hospitalization in the past 12 months due to asthma exacerbation d) Near fatal asthma episode in the past e) Persistent airflow limitation (forced expiratory flow in one second (FEV1) < 60% predicted of normal or daily variability of peak expiratory flow (PEF) > 30%)
7	Weight 20–150 kg
8	Non-smoker
9	The exclusion of other than allergic reactions to inhaled perennial allergens reasons causing severe asthma
10	Exclusion criteria
11	Exacerbations during treatment with OMB if the number is equal to or greater than in the period prior to the treatment year
	Criteria of efficacy not met: a) Response to therapy in GETE less than excellent (complete control of asthma)/good (marked improvement) b) Meeting 2 of the following 3 criteria: • Insufficient improvement in asthma control based on ACQ (change in ACQ score less than or equal to 0.5 points) • Insufficient improvement of the quality of life based on AQLQ or mini AQLQ (change in AQLQ score less than or equal to 0.5 points) • OCS dose reduction of less than or equal 5 mg (only patients treated with OCS before enrolment)
12	Current smoker
13	Noncompliance or poor adherence
14	Initiation of therapy with immunosuppressive drugs, anticancer, infusions of immunoglobulins or other biological agents
15	The occurrence of any contraindications to the use of OMB
16	Pregnant or breastfeeding woman

### Patient population

**Fig. 1:**
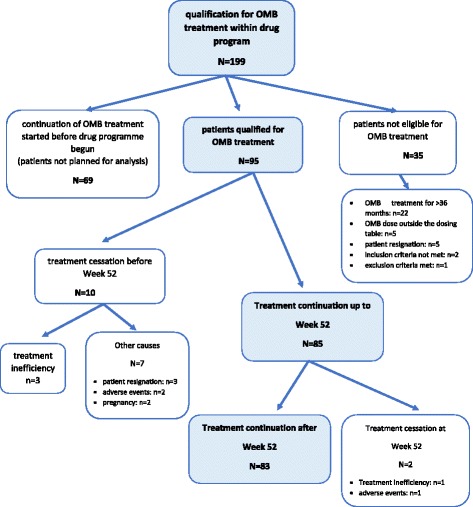
Cohort construction flowchart. From 19.03.2013 to 01.07.2013, 199 applications were submitted to the drug programme. The analysis included a total of 95 patients - only those who initiated therapy with OMB. Sixty-nine patients who had previously received OMB and continued treatment were excluded, as were patients who, for various reasons, were not eligible for this therapy (35 patients). The most common cause of non-eligibility was prior treatment with OMB for a period longer than 36 months (22 patients), as it was initially assumed that the duration of treatment should not exceed 3 years. This was subsequently changed, and now the physician, based on clinical data, decides on the cessation of treatment. The other cause was an inability to determine the dose of the drug by serum total IgE level (IU/mL) measured before the start of treatment and by body weight (kg). Five patients were outside the dosing table, and 2 patients did not meet inclusion criteria; one of them was not allergic, and the other had a tIgE greater than 1500 (IU/mL). Five patients resigned prior to the first dose. Of the 95 patients who started OMB treatment, ten discontinued this therapy before Week 52 (second monitoring point), three of them due to treatment failure observed in Week 16 (first monitoring visit). For the other patients, the treatment was discontinued for reasons such as pregnancy, withdrawal of consent for treatment, and adverse reactions. A group of 85 patients continued the treatment until Week 52, at which time they were evaluated for efficacy and safety. Therapy was then continued in 83 of these patients. Two patients were excluded at this point due to lack of efficacy (one patient) and side effects in the form of generalized erythematous oedema lesions (one patient)

At the time of the analysis, there were 199 patients in the database (Fig. 1).

The criteria for the selection of patients for treatment with OMB (inclusion and exclusion criteria), drug dosing, laboratory tests necessary for qualifying, and a description of the control visits (to monitor the effectiveness and safety of treatment) can be found in the description of the programme (Annex B.44 [10]).

Baseline information on age, sex, asthma status, asthma control, exacerbations, average OCS dose (6 months prior to OMB treatment), tIgE, weight, FEV1, allergy confirmation tests, status of comorbidities, lung function, asthma control and concomitant medications, so as HCU in the year before the beginning of treatment, was collected in an electronic registry. Treatment monitoring visits occurred after 16 and 52 weeks after switching therapy. Patient evaluations included physical examination, spirometry, and response to treatment (Global Evaluation of Treatment Effectiveness - GETE) [5]. Assessment also covered asthma control (based on ACQ scoring) and quality of life (based on miniAQLQ scoring) [11, 12]. Only patients whose GETE was excellent (complete control of asthma) or good (marked improvement) and whose ACQ and miniAQLQ results improved by more than 0.5 points, as well as those whose asthma exacerbations rates were not higher than in the year before therapy, could continue OMB treatment (responders). Other assessments included lung function (FEV1), asthma-related HCU and oral corticosteroid use. Additionally, prior to each OMB administration, asthma control was assessed, and physical examination, vital signs and spirometry were performed as the patients completed questionnaires (ACQ and miniAQLQ).

### Outcomes measure

The analysis of 85 patients who first completed 52 weeks of treatment with OMB was performed with detailed evaluation of the following outcomes:

1. Global Evaluation of Treatment Effectiveness (GETE) [5]
2. OCS dose
3. Asthma control (based on ACQ score)
4. Quality of life (based on miniAQLQ score)
5. Asthma exacerbations (total number of exacerbations including hospitalizations, physician office visits and ED visits due to asthma)

The analysis of clinical outcomes assumed that the patients are responders only if they meet all the following criteria: improvement in quality of life (an increase of greater than 0.5 points in the miniAQLQ score) and asthma control (decrease of greater than 0.5 points in the ACQ score), good or excellent asthma control in GETE and OCS dose reduction not less than 5 mg per day (only patients treated with OCS before enrolment).

### Costs

The cost analysis included the costs of OMB, unscheduled physician visits, emergency department (ED) visits and hospitalizations due to asthma.

The wholesale acquisition cost (WAC) was used for OMB and was 374 (2014 EUR) for one 150-mg vial. The annual cost of treatment with OMB was estimated based on the weight and IgE levels of the patients included. The average dose of OMB was 6645 mg per patient per year, which means that mean annual cost of OMB treatment was 16,565 (2014 EUR).

The cost of exacerbations also included the cost of a physician visit, ED visit and hospitalization, which was based on the average reimbursement of health care resources in Poland, as determined by the public payer (NHF - National Health Fund). The costs of a physician visit, an ED visit and hospitalization due to asthma were 8.4 EUR, 719.42 EUR and 611.0 EUR, respectively. A sensitivity analysis of successful treatment was performed by calculating the costs to achieve a 0,5-point increase in the AQLQ score, a 0,5-point decrease in the ACQ score and a 5-mg decrease in OCS dose.

### Statistical analysis of the study outcomes

General characteristics of the study population, including the percentage of patients who have experienced specific changes in examined end points, were analysed using methods of descriptive statistics. Changes in normally distributed data were analysed using Student’s t-test. Changes in non-normally distributed data were analysed using the Wilcoxon test. All comparisons were two-sided.

In the response to treatment analysis, OCS dose, AQLQ score and ACQ score during visits at week 0, week 16 and week 52 were compared. The Wilcoxon test analysed the reduction in OCS dose. Changes in AQLQ and ACQ were verified by t-test for paired observations. Analysis of asthma exacerbations before and during OMB treatment was performed with the Wilcoxon test.

An alpha level equal to 0.05 / N, where N is the number of post hoc tests performed (according to the Bonferroni correction), was used in the analysis.

Calculations were performed using Statistica 7.1 (StatSoft, Inc).

## Results

### Demographics

A summary of demographic characteristics is reported in Table 2. Thirty eight percent of the patients were male, and 100% were white. The average age was 44.9 years. The average total IgE concentration was 339 IU/ml with a weight of 77.4 kg, and the average dose of OMB counted per individual person, was 554 mg per month. All patients were prescribed two or more asthma controller medications. The first evaluation was after 16 weeks, and the second after 52 weeks of treatment.

**Table 2:** Baseline demographic characteristics

Characteristic	mean	median	range
Age, y	44.90	47.00	13–79
Sex (M/F), n	33/52		
Weight, kg	77.40	74.00	34–140
Total IgE, IU/ml	339.00	294.00	30–1300
Time since diagnosis of asthma, y	23.98	22.00	4–66
FEV1, % predicted at entry	61.78	57.00	26–114
OCS dose, mg at entry	12.59	10.00	5–40
ED visits past 12 mo, n	0.67		0–10
Hospital admissions past 12 mo, n	1.11		0–6
Exacerbation rate past 12 mo, n	6.63		1–30
ACQ at entry	3.60	3.50	1.7–6
AQLQ at entry	2.98	3.10	0–4.9

### Clinical outcomes analysis

Most patients’ response to treatment was good or excellent after 16 and 52 weeks of treatment (95.1 and 93.9% respectively), while at the same time, there was a reduction in the OCS dose (on average by 7.7 mg) (Table 3). Compared with the baseline, the asthma exacerbation rate decreased by 66% during OMB treatment. Additionally, scores for quality of life questionnaires (AQLQ) and asthma control (ACQ) improved. All these changes were statistically significant (*p* < 0,000001).

**Table 3:** Outcome measures (*n* = 85)

Outcome measures	Week 0	Week 16	Week 52
OCS – Mean/Median	12.59 / 10,00	5.68 / 3.7	4.86 / 2.5
Range	[5.00–40.00]	[0.00–40.00]	[0.00–40.00]
*p* value	–	*p* = 0.000000	*p* = 0.000000
AQLQ -Mean/Median	2.98 / 3.10	4.27 / 4.20	4.83 / 4.8
Range	[0.0–4.9]	[1.7–7.0]	[2.5–6.9]
*p* value	–	*p* = 0.000000	*p* = 0.000000
ACQ - Mean/ Median	3.6 / 3.5	2.72 / 2.4	2.15 / 2.10
Range	[1.7–6.0]	[0.4–2.7]	[0.1–5.7]
*p* value	–	*p* = 0.004118	*p* = 0.000000
Asthma exacerbations – Mean	6.63	2.98	1.93
Range	[1.0–30.0]	[0.0–19.5]	[0.0–11.0]
*p* value	–	*p* = 0.000000	*p* = 0.000000

### Cost analysis

The mean asthma treatment cost during the OMB treatment of patients in the programme defined by inclusion and exclusion criteria was 16,781 EUR per patient per year compared with 802 EUR before the therapy.

This significant increase was mainly due to the high price of OMB, as the other costs of health care resources, including hospitalizations and ED and office visits, declined significantly after switching therapy to OMB (from 802 to 216 EUR in the annual assessment). The average annual exacerbation treatment cost is presented in Table 4.

**Table 4:** The average annual exacerbations treatment cost

	Unit Cost (EUR)	At entry				Week 52			
		Patients (*n* = 85)		Per patient		Patients (*n* = 85)		Per patient	
		No of exacerbations	EUR	No of exacerbations	EUR	No of exacerbations	EUR	No of exacerbations	EUR
Physician office visits	8.4	421	14,735.00	4.9529	173.35	157	5495.00	1.8471	64.65
Hospitalizations in Intensive Care Unit (ICU)	719.4	11	12,679.59	0.1294	149.17	0	0.00	0	0.00
ED visits	719.4	57	65,703.33	0.9706	772.98	13	14,984.97	0.1529	176.29
Hospitalizations (all but ICU)	611.0	75	191,100.00	0.8824	2248.24	22	56,056.00	0.2588	659.48

Annual add-on treatment with OMB was also associated with the following:

- a reduction in mean OCS dose by 7.7 mg, wherein the cost of a one-unit decline amounted to 2067.1 EUR.
- an increase in the AQLQ score by 1.86 points, wherein the cost of one unit of growth amounted to 8607.2 EUR
- a reduction in the ACQ score by 1.5 points, wherein the cost of a one-unit decline amounted to 11,024.6 EUR
- a reduction in the number of exacerbations of 4.38 per year. The cost of one exacerbation treated on an outpatient basis and in the hospital amounted to 5144.8 EUR and 12,576.2 EUR, respectively (Table 5).

**Table 5:** The mean change and the cost of the change

The mean change at week 52 of OMB treatment		The cost per unit change (EUR)
OCS dose	−7.72941 (mg)	−2067.3
AQLQ scoring	1.856471 (points)	- 8607.3
ACQ scoring	−1.44941 (points)	−11,024.6
Number of physician office visits^a^	−3.10588	−5144.8
Number of hospitalizations^a^	−1.27059	- 12,576.2
Total number of the physician office visits and hospitalizations^a^	−4.37647	− 3651.2

^a^ only associated with asthma exacerbations

## Discussion

This paper presents the trends in clinical outcomes, quality of life and costs of a one-year treatment with OMB in patients with severe allergic asthma for a cohort of patients who satisfied the inclusion criteria for the initiation of OMB treatment and who successfully responded to OMB according to a set of objective criteria within a drug programme in Poland.

Drug programmes are one method of reimbursement, where patients are integrated into the treatment regimen of a clinical trial (inclusion and exclusion criteria) and then followed in daily practice.

Currently, 42 centres implement the drug programme. The treatment in the drug programme is carried out by physicians experienced in the diagnosis and treatment of severe persistent asthma and is 1restricted to patients prescribed systemic steroids (used chronically or during exacerbations) and for whom all other treatments have failed.

As shown in Poland, patients in this drug programme receive a significant clinical benefit from treatment with OMB, resulting from a reduction in the number of asthma exacerbations, enhanced disease control and improved QoL. There was also a significant decrease in the dose of oral corticosteroids. No clinical improvement was observed in only 3.5% of the severe asthma patients.

Many studies have confirmed the real-world evidence (RWE) of OMB treatment [13–15].

The retrospective analysis of Barnes et al. [13] reported a lower number of exacerbations and enhanced QoL among British patients, consistent with previous studies of OMB. However, improved lung function and a reduction in annual steroid burden was greater in this study than in previous trials.

Findings from the 2-year eXpeRience registry agree with the results from randomized trials, indicating that OMB added to current therapy improves asthma control significantly among patients with uncontrolled persistent allergic asthma [14]. According to a physician’s GETE, 69.9% of patients responded to OMB after 16 weeks. While the percentage of patients with no clinically significant exacerbations was 6.8% during the 12-month pre-treatment period, this value increased to 54.1% at Month 12 and then to 67.3% at Month 24. Compared to the baseline, symptoms and the use of rescue medication decreased by > 50%. Furthermore, maintenance OCS use decreased from 28.6% at the baseline to 16.1 and 14.2% at Months 12 and 24, respectively. Overall, OMB was deemed to have an acceptable safety profile. To summarize, the eXpeRience registry findings are consistent with the results of the clinical trials and suggest that OMB is associated with improved outcomes among patients with uncontrolled persistent allergic asthma. As shown in the Cochrane database published in 2014, OMB was effective in reducing the number of asthma exacerbations and hospitalizations when used with inhaled steroids, as well as during steroid tapering phases of clinical trials [15]. Moreover, OMB was proven to facilitate reducing or withdrawing inhaled steroids more effectively than a placebo.

There is no doubt that the biological treatment of chronic diseases is cost-intensive. Therefore, the decision of the payer and regulator to implement this therapy must be balanced in each country.

In Poland, the drug programme description (with inclusion and exclusion criteria) and final decision on entry into the drug programme is made by the Ministry of Health based on a previous assessment of HTA (in Poland, the Agency for Health Technology Assessment and Pricing - AOTMIT) and the opinion of the Economic Committee, which is involved in negotiating drug prices.

This analysis demonstrated the trends in the costs of treating severe asthma with OMB in Poland within a drug programme. The most cost-intensive occurrence is exacerbation treated in a hospital (12,576.2 EUR), followed by improvement in asthma control (11,024.6 EUR), improvement in QoL (8607.3 EUR), exacerbation treated on an outpatient basis (5144.8 EUR) and lowering the dose of systemic glucocorticosteroids (2067.3 EUR).

It should be emphasized that the analysis presented in this study concerns the direct cost of severe asthma patient treatment with OMB in the drug programme.

This descriptive analysis on the basis of data from the Patient Registry in Poland provides an estimation of costs and outcomes for the defined group of patients with severe asthma, which could be used for the most advanced assessments of severe asthma treatment in Poland.

Unfortunately, the available data from this clearly defined cohort of patients in Poland do not allow one to calculate quality-adjusted life year (QALY) and the incremental cost-effectiveness ratio (ICER) as is recommended in pharmacoeconomic analyses.

There are numerous other publications that refer to QALY or ICER in this area [16–22].

An economic evaluation in a Spanish RWE study was performed from the societal perspective, including direct health costs (resource use and drug treatments) and indirect costs (disease impact on labour productivity) in 2016 Euros (16). When only direct costs were included, the incremental cost-effectiveness ratios (ICERs) were 1487.46 EUR per exacerbation avoided and 5425.13 EUR per 3-point increase in ACT observed. When indirect costs were included, the ICERs were 1130.93 EUR per exacerbation avoided, and 4124.79 EUR per 3-point increase in ACT.

Campbell et al. concluded that adding OMB enhances quality-adjusted life years (QALY) while increasing direct medical costs. Their findings also indicated that using 16-week assessments to guide decisions regarding long-term treatment improves cost-effectiveness [17]. Dal Negro et al. reported that OMB treatment, while improving health-related QoL, increases costs significantly [18].

Youji Oba et al. evaluated the cost-effectiveness of OMB in adults and adolescents with moderate to severe allergic asthma and concluded that from a pharmacoeconomic point of view, considering the high cost and modest efficacy, it would be more beneficial to use OMB in allergic asthmatic patients with poorly controlled symptoms. They emphasized that this therapy would result in a cost reduction only if used among non-smoking patients who were hospitalized 5 or more times or 20 days or longer per year despite using maximal asthma therapy [19].

Van Nooten and Wu et al. assessed OMB treatment to not be cost-effective and reported ICERs of 38.371 EUR and 821.000 USD respectively (ICERs were per QALY gained). Van Nooten et al. concluded that OMB treatment was cost-effective. Moreover, data from the real-life 1-year randomized open-label study (ETOPA) that used Canada as a reference country reported an ICER of 31.209 EUR among patients with severe persistent allergic asthma [20, 21].

The work of Devilde and Oba et al. indicated OMB treatment to be cost-effective when used in patients with severe allergic asthma. These results suggest that to determine the cost-effectiveness of OMB treatment, asthma severity and the risk of asthma exacerbations should be investigated [19, 22].

With a willingness-to-pay (WTP) of 45.000 USD per QALY, OMB treatment was also not cost-effective in adults in Japan, but OMB treatment will continue to be an available treatment option due to its mechanism of action and benefits for severe asthma patients, particularly responders [23]. The cost-effectiveness of OMB treatment may be improved by confining therapy to groups of previously predicted responders and decreasing the price. For paediatric patients in Japan, cost-effectiveness is unclear and requires further study [24].

The interesting study of Faria et al. established the cost-effectiveness of OMB treatment under the list price and the Patient Access Scheme (PAS) discounted price for the UK National Health Service [25]. To assess the long-term cost-effectiveness of OMB treatment, a decision-analytics model was created. QALYs were used to measure the outcomes. Based on data from clinical trials, more specifically, previous hospitalization, maintenance OCS and three or more previous exacerbations, patient subgroups were created. The ICER cost-effectiveness ratio ranged from 30.109 GBP to 57.557 GBP per QALY gained depending on the population considered while using the PAS price; however, incremental cost-effectiveness ratios while using the list price were more than a third higher. The authors suggested that even though the cost-effectiveness of OMB treatment is more favourable under the PAS price, it only offers good value for the money when used in severe asthma patients and requires an optimistic outlook regarding asthma mortality and enhancing health-related QoL.

Recently published Canadian research has shown that OMB treatment was associated with higher costs ($ 1796 Can.) but not with a reduction in clinically important outcomes [26]. The authors suggested that OMB treatment has limited effectiveness in this study population and that future studies should further explore subsets of patients most likely to benefit from OMB therapy.

Considering the high cost of OMB therapy, special attention should be paid to studies assessing the possibility of safe discontinuation of the therapy, prolongation of the intervals between subsequent injections or a reduction in the OMB dose [27, 28]. The OMB dosing regimen should also be observed in real life for both clinical and economic assessment.

## Conclusions

Because treatment with OMB in Poland is carried out in a framework of a drug programme, it is closely monitored, and the data are stored in a registry. Based on this registry, trends in clinical outcomes, quality of life and costs of treatments were assessed in a cohort of patients who satisfied the inclusion criteria for the initiation of OMB treatment and who successfully responded to OMB treatment according to a set of objective criteria.

The clinical outcomes were highly improved. At the same time, the costs of treatment increased, mainly due to high OMB costs. Other costs associated with a decreased number of hospitalizations and ED and office visits and a reduction in OCS doses declined. It should be noted that even among patients who successfully respond to OMB, the cost of therapy remain high and is far from being offset by the reduction in adverse clinical outcome.These descriptive data can be used to further investigate and define patients who benefit the most from OMB treatment in clinical practice.

## Abbreviations

ACQ: Asthma control questionnaire
AOTMIT: Agency for Health Technology Assessment
AQLQ: Asthma quality of life questionnaire
ED: Emergency Department
FEV1: Forced expiratory flow in one second
GETE: Global Evaluation of Treatment Effectiveness
GINA: Global Initiative for Asthma
HCU: Health care utilization
ICERs: Incremental cost-effectiveness ratios
ICS: Inhaled corticosteroids
LABA: Long-acting beta-agonists
LTRA: Leukotriene receptor antagonists
NHF: National Health Fund
OCS: Oral steroid
OMB: Omalizumab
PAS: Patient access scheme
QALY: Quality-adjusted life years
QoL: Quality of life
RWD: Real-world data
RWE: Real-world evidence
SAMA/LAMA: Short- or long-acting muscarinic antagonists
tIgE: Total IgE
WAC: Wholesale acquisition cost
WTP: Willingness-to-pay
